# Supplementary material for: EGFR/ARF6 regulation of Hh signalling stimulates oncogenic Ras tumour overgrowth
Source: Nat Commun. 2017 Mar 10;8:14688. doi: 10.1038/ncomms14688 (PMC5353614; doi:10.1038/ncomms14688)
Supplement: Supplementary Information — Supplementary Figures and Supplementary Note. [file ncomms14688-s1.pdf]

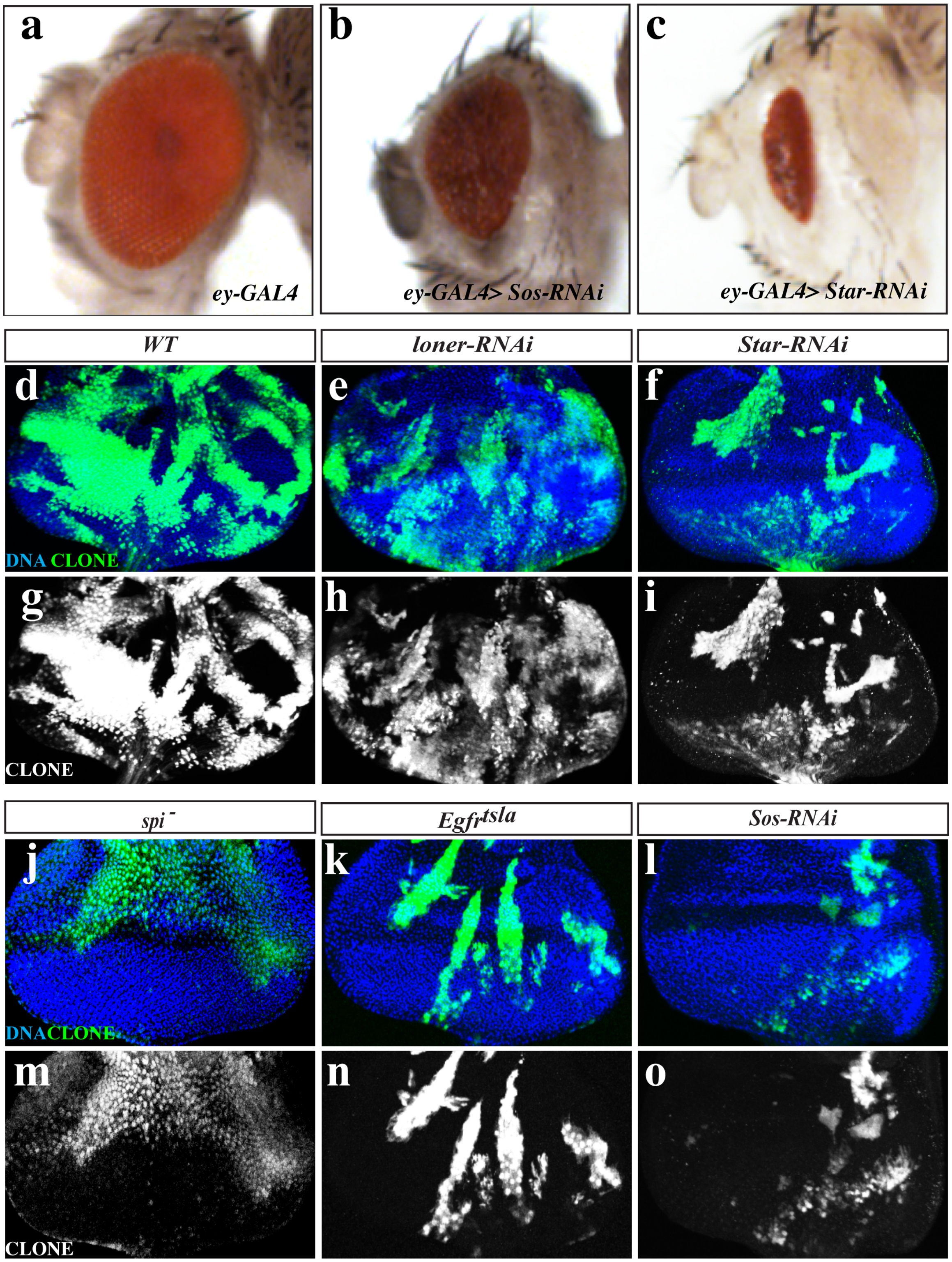

a
ey-GAL4
b
ey-GAL4> Sos-RNAi
c
ey-GAL4> Star-RNAi
WT
loner-RNAi
Star-RNAi
d
e
f
DNA CLONE
g
h
i
CLONE
spi -
Egfr tsla
Sos-RNAi
j
k
l
DNACLONE
m
n
o
CLONE

Supplementary Figure 1.

Growth analysis.

a-c) Adult eyes expressing *ey-GAL4* alone (**a**) or co-expressing *Sos-RNAi* (**b**) or *Star-RNAi* (**c**).

d-o) Images of third-instar eye discs containing the clones (green) of the indicated genotype and stained with DAPI. Bottom panels (**g-i** and **m-o**) show corresponding clone (GFP) channel alone.

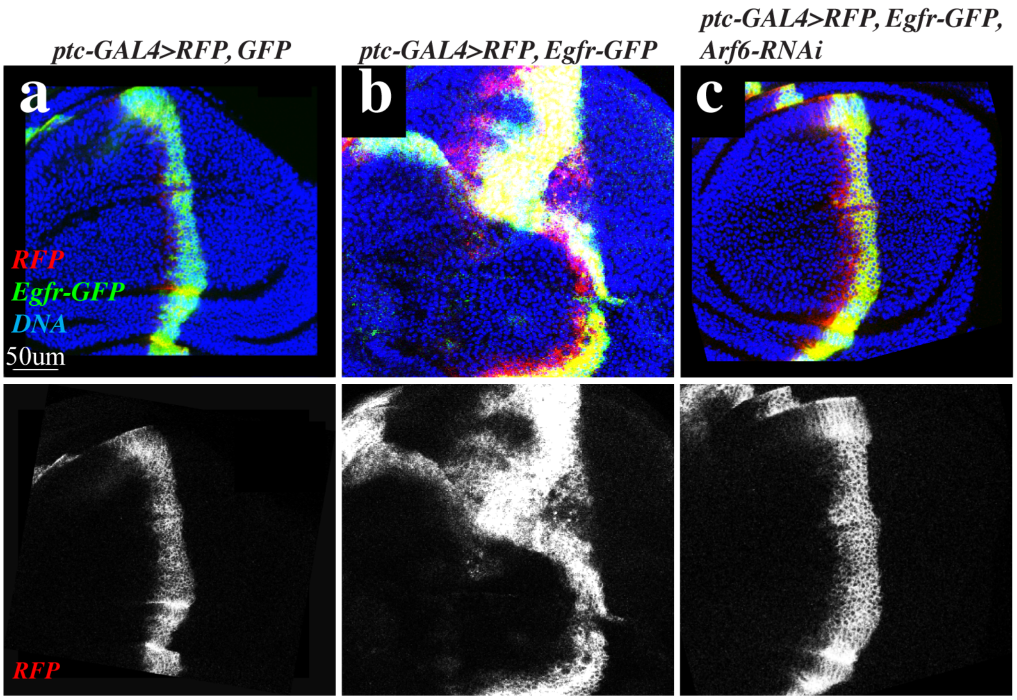

Supplementary Figure 2.

Arf6 mediates Egfr signaling growth control.

a-c) Images showing *patched-GAL4* driven co-expression of RFP with GFP (**a**) or Egfr-GFP (**b**) or with Egfr-GFP and *Arf6-RNAi* (**c**). Egfr-GFP causes an overgrowth and malformation of the *patched* domain (**b**). Co-expression of *Arf6-RNAi* suppresses Egfr-GFP-mediated overgrowth.

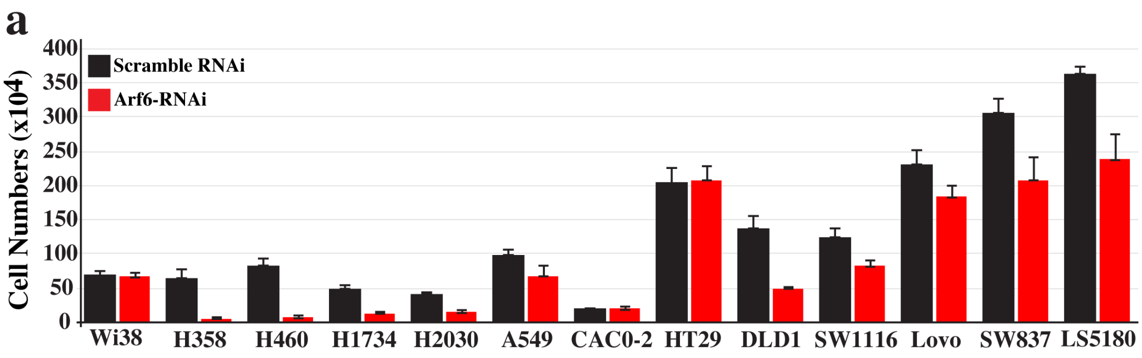

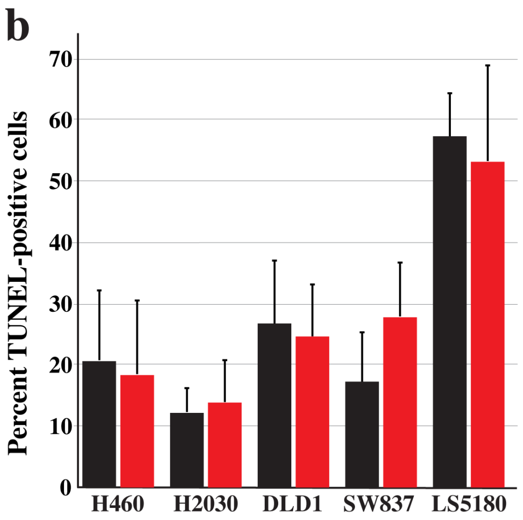

Supplementary Figure 3.

Arf6 knockdown suppresses the growth of cancer cells independent of apoptosis.

Quantitation of cell numbers 48 hours after cancer cells were transfected with either Scramble (control) or Arf6 RNAi.

Cell death analysis of Arf6 RNAi responsive cancer cells from (**a**). The percentage of TUNEL-positive cancer cells 48 hours after transfection with either Scramble (control) or Arf6 RNAi are shown. Across all cell lines examined, *P* values between experimental and control RNAi-treated cells were greater than 0.2.

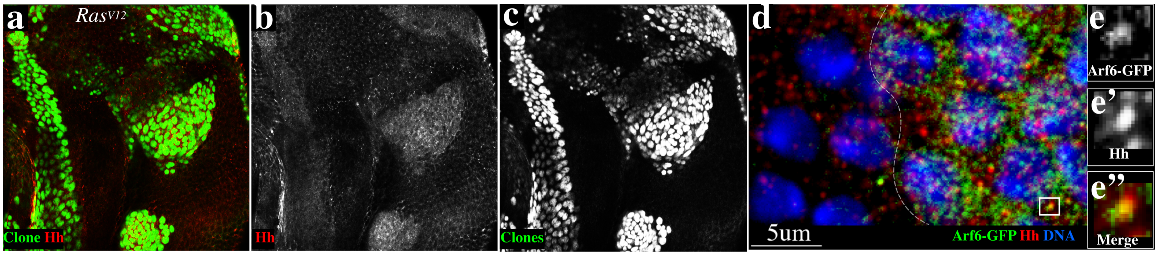

Supplementary Figure 4.

Hh overexpression and co-localization with Arf6-GFP in *Ras*^{V12} cells.

a-c) Eye disc showing *Ras*^{V12} clones (green) and stained against Hh (red).

d-e’’) *Ras*^{V12} clones generated in tissues where Arf6-GFP expression is under endogenous control and counterstained with DAPI (blue) and anti-Hh (red) antibodies. The dotted white line denotes the clone (right side) boundary. Individual and merge channels of the boxed area (**d**) are shown in (**e-e’’**).

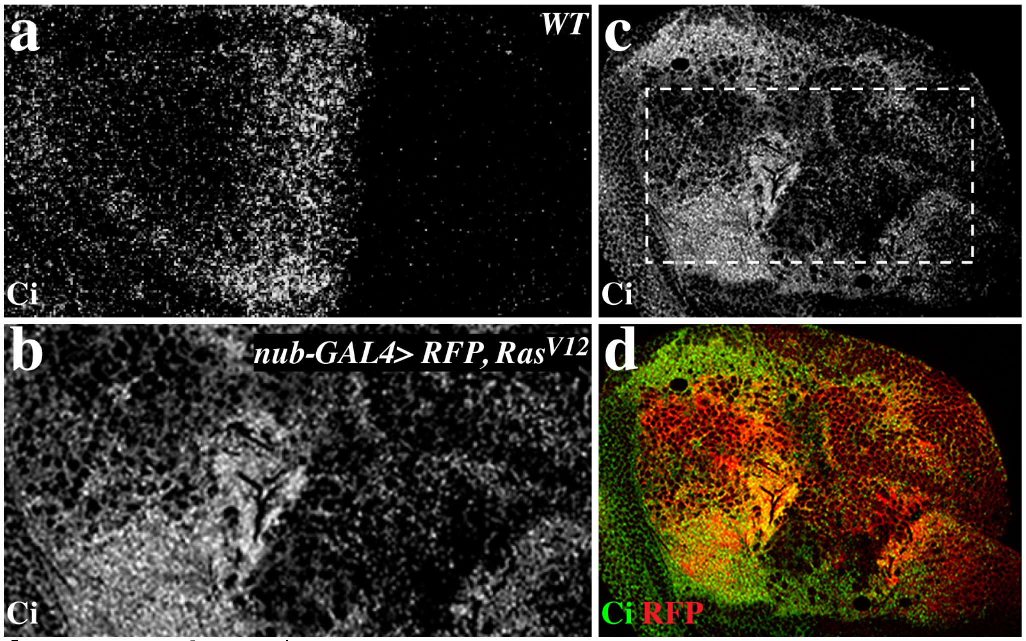

Supplementary Figure 5.

Ras^{V12} **activates Hh signaling in the wing.**

a-**d**) Images showing the pouch region of wing discs stained against full-length Ci to assess Hh signaling. Ci activation is restricted to the anterior compartment of wild-type discs (**a**). In contrast, expression of *Ras^{V12}* using *nub-GAL4* leads to elevated ci levels throughout the wing pouch (RFP-labeled, panel **d**), including in the posterior compartment of the wing (**b**). A lower magnification is presented in (**c**). The boxed area represents the region of disc shown in (**b**).

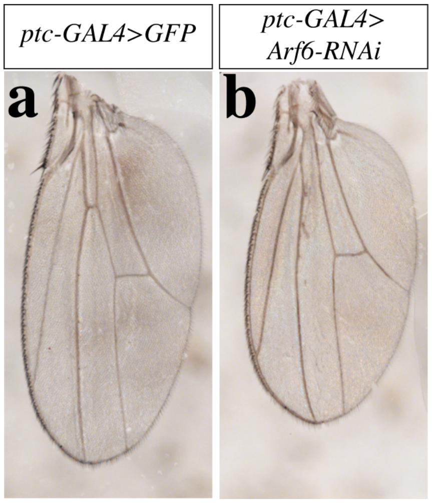

Supplementary Figure 6.

Arf6 RNAi knockdown reduces wing size and causes vein defects.

a and **b**). *ptc-GAL4* expression of GFP (**a**) or *Arf6-RNAi* (**b**). *Arf6-RNAi* causes wing size reduction and loss of the anterior cross-vein.

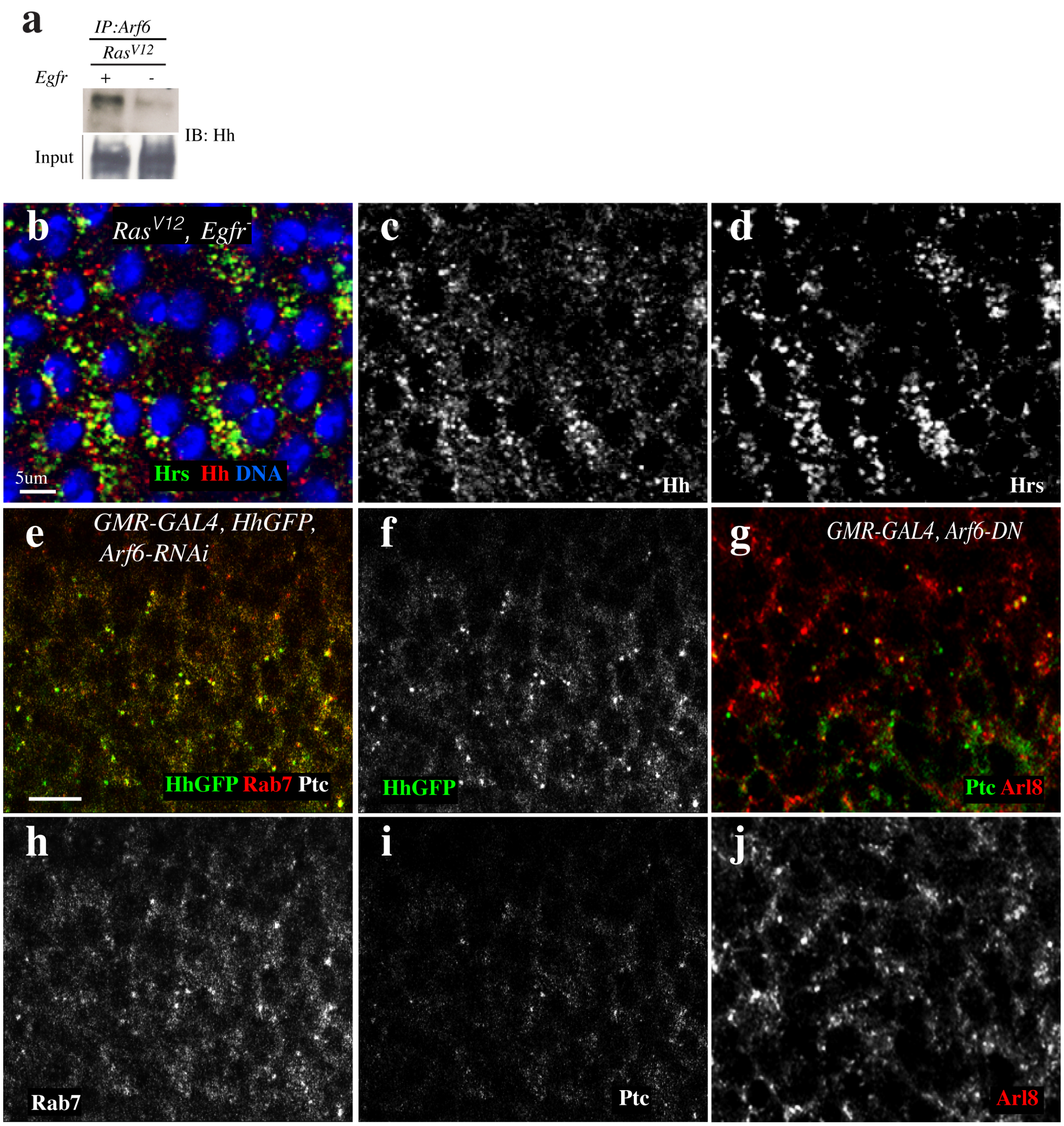

Supplementary Figure 7.

***EgfrR* or *Arf6* knockdown causes Hh to localize to degradation vesicles.**

Arf6 Immunoprecipitation experiments using lysate derived from third-instar discs harboring *Ras^{V12}* or *Ras^{V12}*, *$Egfr^-$* double mutant clones blotted against Hh. Pre-precipitation lysate were blotted for total Hh protein. Hh was equally abundant in *Ras^{V12}* or *Ras^{V12}*, *$Egfr^-$* double mutant tissues lysates but the *$Egfr^-$* mutation interfered with Arf6's ability to interact with Hh.

b-d) *Ras*^{V12}*, Egfr*$^{-}$ double mutant cells stained with DAPI (blue), anti-Hh (red), and anti-Hrs (green). Individual channels for Hh and Hrs are shown in (**c**) and (**d**), respectively.

e) Image showing anterior/posterior region of the eye disc co-expressing of *Arf6-RNAi* and *Hh-GFP* using *GMR-GAL4* and stained with anti-patched and anti-Rab7 antibodies. Individual Hh-GFP, Rab7, and patched channels are respectively shown in (**f)**, (**h)**, and (**i**).

g) Image of eye disc expressing of *Arf6-DN* under *GMR-GAL4* and stained against patched and Arl8. The corresponding Arl8 channel is shown alone in (**j**).

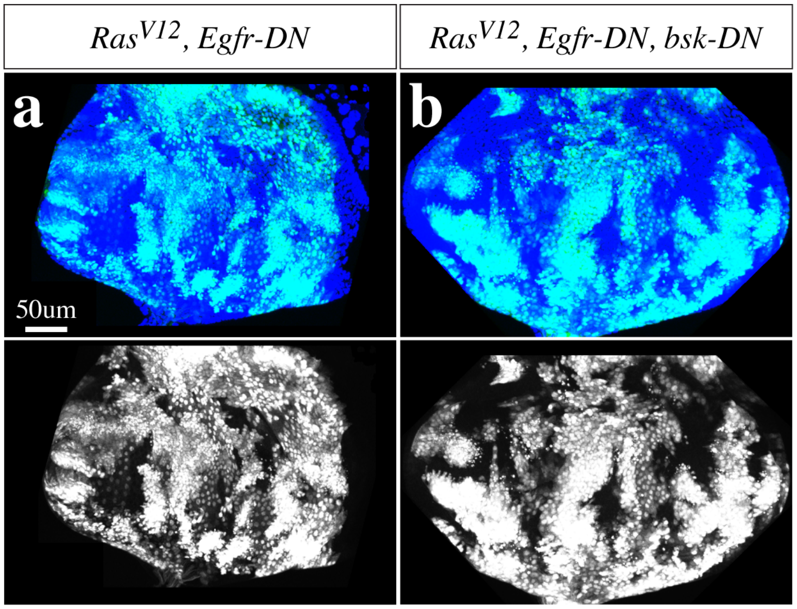

Supplementary Figure 8.

JNK-inhibition fails to rescue the growth of *Ras^{V12}*, *Egfr-DN*

a and **b**) Images of third-instar eye disc containing either *Ras^{V12}, Egfr-DN* double (**a**) or *Ras^{V12}, Egfr-DN, bsk-DN* triple (**b**) mutant clones. Inhibition of JNK signaling by expressing a JNK dominant-negative version (*bsk-DN*) shows no detectable effect on clone growth.

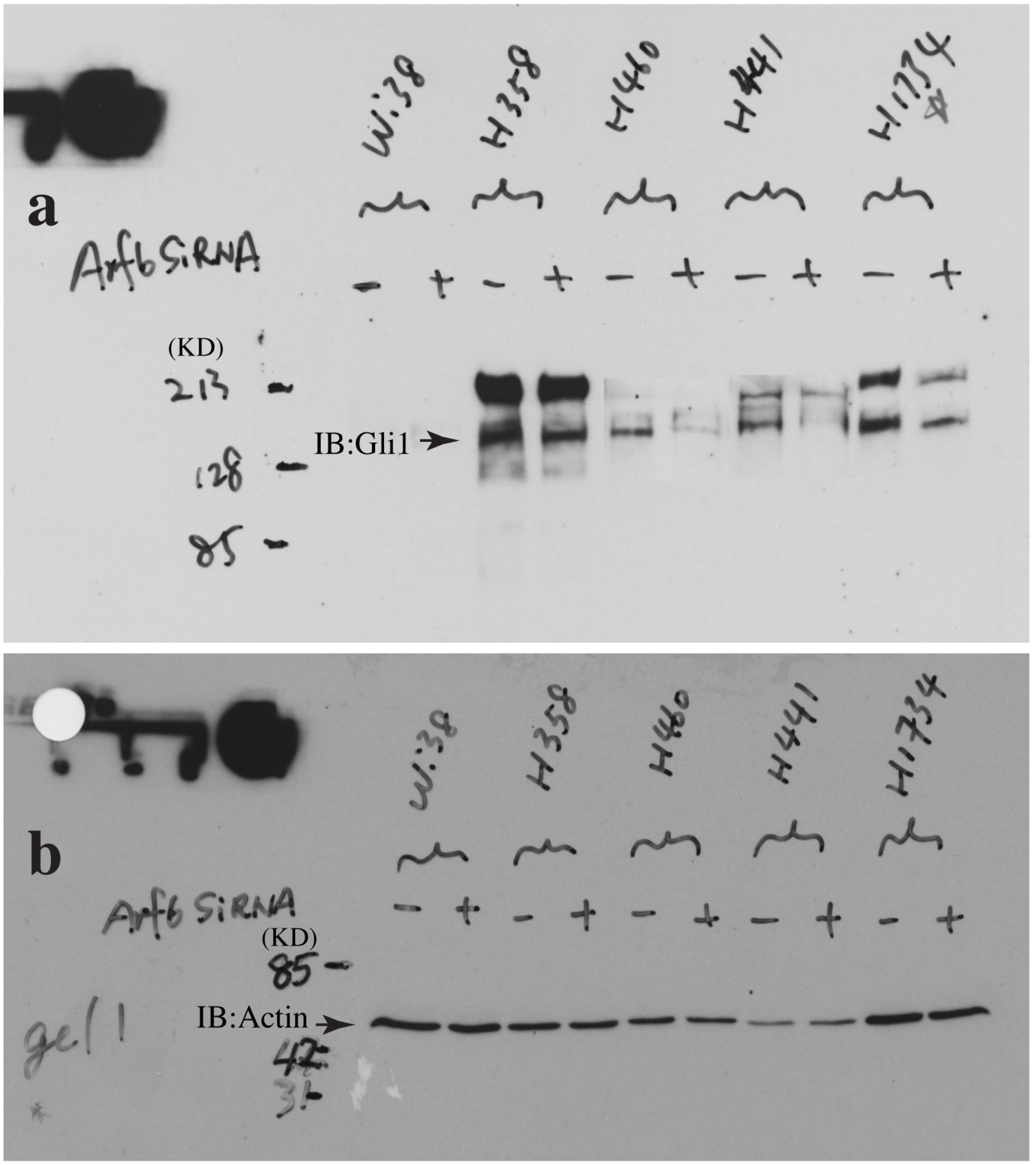

Supplementary Figure 9.

Arf6 controls Hh signaling in lung cancer cells.

Related to figure 4 q in the main text. Uncropped scans showing Western blotting of various lung cancer cell lines treated with scramble or Arf6 RNAi and blotted with anti-Gli1 to detect Gli1 protein abundance (a, arrow) and hence Hh signaling levels

or blotted with anti-actin (b, arrow) as a loading control. Arf6-RNAi causes a reduction in Gli1 levels across lung cancer cell lines.

Supplementary Note 1.

Detailed genotypes.

Figure 1

1a: *y w,ey-Flp1/+; act>y+>GAL4,UAS-GFP.S65T/+; FRT82B,tub-Gal80/FRT82B*

1b, 1k, 1l, 1o, and 1q: *y w,ey-Flp1/+; act>y+>GAL4,UAS-GFP.S65T/+; FRT82B,tub Gal80/FRT82B,UAS-Ras*^{V12}

1c, 1m, 1n, 1p, and 1r: *y w,ey-Flp1/+;/+; FRT42D,tub-Gal80/FRT42D,Egfr*^{topCo}*;act>y+>GAL4,UAS-GFP.S65T/+*

1d: *y w,ey-Flp1/+;/+; FRT42D,tub- Gal80/FRT42D,Egfr*^{topCo}*;act>y+>GAL4,UAS-GFP.S65T/ UAS-Ras*^{V12}

1e: *y w,ey-Flp1/+; act>y+>GAL4,UAS-GFP.S65T/UAS-Egfr-DN+; FRT82B,tub-Gal80/FRT82B, UAS-Ras*^{V12}

Figure 2

2a-c: *y w,ey-Flp1/+; act>y+>GAL4,UAS-GFP.S65T/+; FRT82B,tub- Gal80/ FRT82B,UAS-Ras*^{V12}

2d: *yw,ey-Flp1/+;tub-GAL80,FRT40A/FRT40A, spi*$^{-}$*;act>y+>GAL4,UAS-GFP.S65T/UAS- Ras*^{V12}

2e: *yw,ey-Flp1/+;tub-GAL80,FRT40A/FRT40A, UAS-Ras*^{V12}*;act>y+>GAL4,UAS-GFP.S65T/UAS-Star-RNAi*

2f: *y w,ey-Flp1/+;/+; FRT42D,tub-Gal80/FRT42D,Egfr*^{tsla}*;act>y+>GAL4,UAS-GFP.S65T/UAS-Ras*^{V12}

2g: *yw,ey-Flp1/+;tub-GAL80,FRT40A/FRT40A, UAS-Ras*^{V12}*;act>y+>GAL4,UAS-GFP.S65T/UAS-Sos-RNAi*

2h: *yw,ey-Flp1/+;tub-GAL80,FRT40A/FRT40A, ras*^{c40A}*;act>y+>GAL4,UAS-GFP.S65T/+*

2i: *yw,ey-Flp1/+;tub-GAL80,FRT40A/FRT40A, ras*^{c40A}*;act>y+>GAL4,UAS-GFP.S65T/UAS-Ras*^{V12}

Figure 3

3a: *y w,ey-Flp1/+; act>y+>GAL4,UAS-GFP.S65T/+; FRT82B,tub-Gal80/FRT82B,UAS-Ras*^{V12}

3b: *y w,ey-Flp1/+; act>y+>GAL4,UAS-GFP.S65T/UAS- Arf6-RNAi; FRT82B,tub-Gal80/FRT82B*

3c: *y w,ey-Flp1/+; act>y+>GAL4,UAS-GFP.S65T/UAS- Arf6-RNAi; FRT82B,tub-Gal80/ FRT82B,UAS-Ras*^{V12}

3d: *yw,ey-Flp1/+;tub-GAL80,FRT40A/FRT40A,UAS-Ras*^{V12}*;act>y+>GAL4,UAS-GFP.S65T/UAS-steppke-RNAi*

3e: *y w,ey-Flp1/+; act>y+>GAL4,UAS-GFP.S65T/UAS-loner-RNAi; FRT82B,tub-Gal80/ FRT82B,UAS-Ras*^{V12}

Figure 4

4a: *(WT) y w,ey-Flp1/+; act>y+>GAL4,UAS-GFP.S65T/+; FRT82B,tub*

Gal80/FRT82B and (Ras^{V12}*) y w,ey-Flp1/+; act>y+>GAL4,UAS-GFP.S65T/+; FRT82B,tub Gal80/FRT82B, UAS-*Ras^{V12}

4b, 4c, and 4n: y w,ey-Flp1/+; act>y+>GAL4,UAS-GFP.S65T/+; FRT82B,tub Gal80/FRT82B,UAS-Ras^{V12}

4d: *y w,ey-Flp1/+; act>y+>GAL4,UAS-GFP.S65T/UAS-ARF6-RNAi; FRT82B,tub-Gal80/ FRT82B,UAS-*Ras^{V12}

4e: *yw,ey-Flp1/+;tub-GAL80,FRT40A/FRT40A,* spi^{-}*;act>y+>GAL4,UAS-GFP.S65T/ UAS-*Ras^{V12}

4f: *yw,ey-Flp1/+;/+; FRT42D,tub-Gal80/FRT42D,*$Egfr^{topCo}$*;act>y+>GAL4,UAS-GFP.S65T/UAS-*Ras^{V12}

4g and 4j: *yw hsp70-Flp/+; act-*y^{+}*-GAL4 UAS-GFP/+; UAS-Arf6-RNAi/+*

4h: *Oregon R*

4k: *ap-GAL4,UAS-myrRFP/+;UAS- Arf6-RNAi/+*

4l: *ptc-GAL4,UAS-myrRFP/UAS-GFP*

4m: *ptc-GAL4,UAS-myrRFP/UAS-Arf6-DN*

4o: *yw,ey-Flp1/+;tub-GAL80,FRT40A/FRT40A,UAS-*Ras^{V12}*;act>y+>GAL4,UAS-GFP.S65T/*Hh^{fse}

4p: *yw,ey-Flp1/+;tub-GAL80,FRT40A/FRT40A,UAS-*Ras^{V12}*;act>y+>GAL4,UAS-GFP.S65T/UAS-ci-RNAi*

Figure 5

5b: *y w,ey-Flp1/+; act>y+>GAL4,UAS-GFP.S65T/+; FRT82B,tub-Gal80/FRT82B,UAS-*Ras^{V12}

5c, 5c’, and 5g: *UAS-Arf6-DN/UAS-Hh-GFP;* Ras^{V12}*/GMR-GAL4*

5d and 5h: *UAS-Rab7-GFP/+; UAS-*Ras^{V12}*/GMR-GAL4*

5f: *y w,ey-Flp1/+; act>y+>GAL4,UAS-GFP.S65T/UAS- Arf6-RNAi; FRT82B,tub-Gal80/FRT82B,UAS-*Ras^{V12}

5i and 5m: *UAS-Arf6-DN/UAS-Rab7-GFP; UAS-*Ras^{V12}*/GMR-GAL4*

5j and 5n: *UAS-Hh.N.GFP/UAS- Arf6-RNAi;UAS-*Ras^{V12}*/GMR-GAL4*

5k and 5o: *UAS-Hh.N.GFP/+;UAS-*Ras^{V12}*/GMR-GAL4*

5l and 5p: *UAS-Arf6-RNAi/GMR-GAL4*

Supplemental Figures

Figure 1

1a: *ey-GAL4/+*

1b: *ey-GAL4/+;UAS-Sos-RNAi/+*

1c: *ey-GAL4/+;UAS-Star-RNAi/+*

1d and 1g: *y w,ey-Flp1/+; act>y+>GAL4,UAS-GFP.S65T/+; FRT82B,tub-Gal80/ FRT82B*

1e and 1h: *y w,ey-Flp1/+; act>y+>GAL4,UAS-GFP.S65T/UAS-loner-RNAi; FRT82B,tub-Gal80/ FRT82B*

1f and 1l: *yw,ey-Flp1/+;tub-GAL80,FRT40A/FRT40A;act>y+>GAL4,UAS-GFP.S65T/UAS-Star-RNAi*

1j and 1m: *yw,ey-Flp1/+;tub-GAL80,FRT40A/FRT40A,* spi^{-}*;act>y+>GAL4,UAS-GFP.S65T/+*

6k and 6n: *y w,ey-Flp1/+;/+; FRT42D,tub-Gal80/FRT42D,*$Egfr^{tsla}$*;act>y+>GAL4,UAS-GFP.S65T/+*

6l and 6o: *yw,ey-Flp1/+;tub-GAL80,FRT40A/FRT40A;act>y+>GAL4,UAS-GFP.S65T/UAS-Sos-RNAi*

Figure 2

2a: *ptc-GAL4, UAS-myr-RFP/+; UAS-GFP/+*

2b: *ptc-GAL4, UAS-myr-RFP/EGFR-GFP; +/+*

2c: *ptc-GAL4, UAS-myr-RFP/Egfr-GFP; UAS-Arf6-RNAi/+*

Figure 4

4a-c: *y w,ey-Flp1/+; act>y+>GAL4,UAS-GFP.S65T/+; FRT82B,tub-Gal80/FRT82B,UAS-Ras*^{V12}

4d-e”: *y w,ey-Flp1/+; act>y+>GAL4,UAS-GFP.S65T/ARF6-GFP; FRT82B,tub-Gal80/FRT82B,UAS-Ras*^{V12}

Figure 5

5a: OregonR

5b-d: *nub-GAL4, UAS-src-RFP/+; UAS-Ras*^{V12}*/+*

Figure 6

6a: *ptc-GAL4, UAS-myr-RFP/UAS-GFP*

6b: *ptc-GAL4, UAS-myr-RFP/UAS- Arf6-RNAi*

Figure 7

7b-4d: *y w,ey-Flp1/+;/+; FRT42D,tub- Gal80/FRT42D,Egfr*^{topCo}*;act>y+>GAL4,UAS-GFP.S65T/ UAS-Ras*^{V12}

7e, 7f, 7h, and 7i: *UAS-Hh-GFP/+; GMR-GAL4/UAS- Arf6-RNAi*

7g and 7j: *UAS- Arf6-DN*/+; *GMR-GAL4/+*

Figure 8

8a: *y w, ey-Flp1/+; act>y+>GAL4,UAS-GFP.S65T/UAS-Egfr-DN+; FRT82B,tub-Gal80/FRT82B, UAS-Ras*^{V12}

8b:*UAS-BskDN/+;ey-FLP5, Act>y+>Gal4, UAS-GFP/UAS-Egfr-DN;FRT82B, Tub-Gal80/ FRT82B, UAS-Ras*^{V12}
